# Tonsillar Tfh cells contribute to the pathogenesis of IgA nephropathy in collaboration with memory B cells

**DOI:** 10.3389/fimmu.2026.1848238

**Published:** 2026-09-03

**Authors:** Taiki Sugaya, Ippei Ikegami, Ryuta Kamekura, Akira Yorozu, Hiroshi Sakamoto, Ayumi Tatekoshi, Masanobu Tanemoto, Takeshi Ohyu, Shotaro Shirato, Ken-ichi Takano, Shingo Ichimiya

**Affiliations:** 1 Department of Immunology, Research Institute for Immunology, Sapporo Medical University School of Medicine, Sapporo, Japan; 2 Department of Otolaryngology-Head and Neck Surgery, Sapporo Medical University School of Medicine, Sapporo, Japan

**Keywords:** anti-Tn antibody, Gd-IgA, IgA nephrology, memory B cell, Tfh (follicular helper T cells), tonsillectomy

## Abstract

**Introduction:**

Immunoglobulin A nephropathy (IgAN) is a chronic glomerular disease characterized by mesangial IgA deposition, which can progress to end-stage renal failure. Tonsillectomy is used as a therapeutic intervention to slow disease progression and provide early clinical benefit. However, the immune mechanisms within the palatine tonsils that contribute to IgAN pathogenesis remain incompletely understood.

**Methods:**

To define the tonsillar immune environment that regulates pathological antibody production, we analyzed T and B lymphocyte subsets in relation to renal function. We also conducted functional studies to evaluate the production of galactose-deficient IgA (Gd-IgA) and anti-Tn (GalNAc-Ser/Thr) antibodies, both of which are implicated in the formation of pathogenic immune complexes.

**Results:**

Interfollicular T follicular helper (IF-Tfh) cells (CD3^+^CD4^+^CD8^-^PD-1^lo^CXCR5^lo^) were significantly expanded in IgAN tonsils compared with disease controls and strongly correlated with clinical markers of renal abnormalities. Transcriptomic analysis of IF-Tfh cells from IgAN tonsils revealed a distinct gene expression profile enriched for effector memory T cell–like features associated with kidney impairment. Functional studies demonstrated that IF-Tfh cells potently promoted class-switched memory B cells to produce Gd-IgA1 and anti-Tn (GalNAc-Ser/Thr) antibodies.

**Conclusion:**

These findings suggest that an IF-Tfh cell–dominant immune environment drives IgAN pathogenesis and may represent a target for noninvasive therapeutic approaches.

## Introduction

Immunoglobulin A nephropathy (IgAN) is the leading cause of primary glomerulonephritis worldwide and is characterized by deposition of IgA-containing immune complexes in the mesangial regions of the glomeruli (1, 2). Although the severity of histopathologic injury varies among patients because of underlying genetic and environmental heterogeneity, IgAN typically follows a slowly progressive course. Approximately one-third of affected individuals develop chronic kidney disease, and some eventually progress to end-stage renal failure. Therefore, early prevention and intervention are critical, and current treatment strategies focus on symptom control to slow disease progression (3). General management includes optimizing blood pressure and using corticosteroids or other immunosuppressive agents to preserve nephron function and limit further damage.

IgA1 with an aberrantly O-glycosylated hinge region, known as galactose-deficient IgA1 (Gd-IgA1), is found in the serum of affected patients and within glomerular immune deposits, where it promotes inflammation, activates the complement cascade, and ultimately contributes to glomerular scarring (2, 4–6). Although Gd-IgA1 lacks clear antigen specificity, it displays the Thomsen-nouveau (Tn) antigen (GalNAcα1-O-Ser/Thr) as an exposed galactose-deficient epitope (5). This epitope is recognized by anti-Tn antibodies, a subset of anti-glycan autoantibodies that are elevated in the sera of patients with IgAN (7). The interaction between Gd-IgA1 and these antibodies promotes the formation of pathogenic immune complexes, thereby contributing to kidney inflammation. Current therapeutic strategies target various pathogenic processes to reduce glomerular injury caused by Gd-IgA1 immune complex deposition, with several demonstrating efficacy in clinical trials (8–11). Despite significant progress, the precise pathophysiological mechanisms underlying IgAN remain incompletely defined and continue to hinder the development of optimal disease management strategies.

IgA plays a fundamental role in mucosa-associated lymphoid tissue (MALT) by protecting against pathogens and maintaining homeostasis with commensal flora (12, 13). Thus, MALT structures such as nasopharynx-associated lymphoid tissue (NALT) and gut-associated lymphoid tissue (GALT) have received considerable attention in efforts to elucidate the etiology of IgAN. As a key component of NALT, the palatine tonsils are central to mucosal immunity in the oral cavity and have been implicated in the pathogenesis of IgAN (14, 15). The clinical efficacy of tonsillectomy in preventing progression to chronic renal failure underscores the need to clarify how tonsillar immunity contributes to the onset of IgAN. Recent advances involving a targeted-release formulation of budesonide designed to act in the small intestine further support a pivotal role for GALT in IgAN (16, 17). Together, these observations indicate that both NALT and GALT contribute to the development of IgAN. Because of practical challenges in studying clinical specimens from intestinal sites, such as mucosal tissues and Peyer’s patches, surgically resected tonsils are widely regarded as a valuable source for investigating the involvement of NALT-resident immune cells in disease pathogenesis. Notably, Gd-IgA1 has been detected in the tonsils of affected patients (18), and research has increasingly focused on characterizing the immune landscape within IgAN tonsils to provide deeper insight into IgAN pathophysiology.

To elucidate the immune environment of IgAN tonsils, we analyzed tonsillar lymphocytes, including T and B cell subsets, with particular focus on T follicular helper (Tfh) cells and their roles in immunoglobulin production and immune complex formation (19–21). We found that IgAN tonsils display distinct lymphocyte profiles associated with renal dysfunction, as reflected by clinical serum markers. Remarkably, IgAN tonsils preferentially harbor interfollicular Tfh (IF-Tfh) cells, which localize to interfollicular regions of lymphoid follicles (22, 23). IF-Tfh cells from IgAN tonsils exhibited transcriptomic characteristics resembling effector memory T cells, in contrast to those from recurrent tonsillitis. This profile suggests the persistent presence of aberrant IF-Tfh cells in IgAN. Furthermore, *in vitro*, IF-Tfh cells derived from IgAN tonsils promoted class-switched memory B cells to produce Gd-IgA1 and anti-Tn antibodies. These findings underscore the potential of targeting IF-Tfh–mediated pathways as disease-specific interventions to prevent the formation of nephritogenic immune complexes.

## Materials and methods

### Clinical specimens

The study populations are summarized in **Supplementary Tables 1**–**6** and **10**. Tonsil samples were obtained from patients who underwent tonsillectomy for IgAN (n = 54), RT (n = 43), PPP (n = 29), or OSAS (n = 47) at Sapporo Medical University Hospital or JR Sapporo Hospital. Among the patients with IgAN, one had concomitant Crohn’s disease and was treated with adalimumab, while another had recurrent IgAN after kidney transplantation and was receiving tacrolimus, mycophenolate mofetil, everolimus, and methylprednisolone. Clinical information, including gender, age, and laboratory test results, was collected for each participant. The eGFR was calculated using an equation specific to the Japanese population: eGFR (mL/min/1.73 m^2^) = 194 × (serum creatinine)^−1.094^ × (age)^−0.287^. For female patients, the resulting value was multiplied by 0.739 (24). Exclusion criteria for correlation analysis in patients with IgAN were age < 18 years and eGFR < 30 mL/min/1.73 m^2^. Peripheral blood samples from patients and healthy volunteers were collected before initiation of any treatment. Tonsillar tissues were dissected and gently dissociated to isolate lymphocytes for subsequent analyses. The protocol for patient sample collection and analysis was approved by the Institutional Review Board of Sapporo Medical University Hospital (IRB approval #25–39 and #292-83). Written informed consent was obtained from all participants in accordance with the Declaration of Helsinki. All relevant ethical regulations for research involving human participants were followed.

### Flow cytometric analysis and cell sorting

Single-cell suspensions were generated from tonsillar tissues by passing cells through a 70-µm cell strainer (Corning, catalog no. 352350), followed by density-gradient centrifugation with Lympholyte-H cell separation reagent (Cedarlane, catalog no. CL5020-R). Cells were stained on ice for 1 hour with fluorochrome-conjugated monoclonal antibodies diluted in FACS buffer (PBS containing 0.5% bovine serum albumin and 2 mM EDTA). After washing, cells were analyzed or sorted on a FACS Canto II or FACS Aria III (BD Biosciences) using the MagniSort Human CD19 Positive Selection Kit (Thermo Fisher Scientific, catalog no. 8802-6833-74) or the CD4 T Cell Enrichment Kit (Thermo Fisher Scientific, catalog no. 8804-6811-74). Singlets were identified by doublet discrimination in all experiments. Flow cytometry data were analyzed with FACSDiva software (BD Biosciences, version 8.0) and FlowJo software (BD Biosciences, version 10.10.0). The purity of FACS-sorted cell populations was confirmed to be greater than 95% by reanalysis on the FACS Aria III. A complete list of fluorochrome-conjugated antibodies used in this study is provided in **Supplementary Table 11**.

### *In vitro* coculture assays

Sorted swBm cells (CD19^+^IgD^−^CD27^+^CD24^+^CD38^−^) and unswBm cells (CD19^+^IgD^+^CD27^+^) were cocultured at a 1:1 ratio (5 × 10^4^ cells per population) with either naive T cells (CD4^+^CD19^−^CD45RA^+^), IF-Tfh cells (CD4^+^CD19^−^CD45RA^−^PD-1^lo^CXCR5^lo^), or GC-Tfh cells (CD4^+^CD19^−^CD45RA^−^PD-1^hi^CXCR5^hi^) isolated from tonsils. Cocultures were established in AIM-V serum-free medium (Thermo Fisher Scientific, catalog no. 0870112DK) supplemented with anti-CD3 antibody (2 μg/mL, Miltenyi Biotec, catalog no. 130-093-377), anti-CD28 antibody (2 μg/mL, Miltenyi Biotec, catalog no. 130-093-375), recombinant human CD40 ligand (1 μg/mL, R&D Systems, catalog no. 2706-CL-025/CF), and penicillin-streptomycin-amphotericin B suspension (1:100 dilution, Fujifilm Wako Pure Chemical Corporation, catalog no. 161-23181). Cells were cultured for 10 days in 96-well U-bottom plates at 37 °C in a humidified atmosphere containing 5% CO_2_. After incubation, culture supernatants were collected and analyzed by enzyme-linked immunosorbent assay (ELISA).

### ELISA

To measure Gd-IgA1 levels, coculture supernatants diluted 1:2 were analyzed using a Gd-IgA1 assay kit (IBL, catalog no. 27600) according to the manufacturer’s instructions. For quantifying anti-Tn immunoglobulin titers, 96-well ELISA plates (Thermo Fisher Scientific, catalog no. 468667) were coated with 10 µg/mL Tn antigen (Merck, catalog no. 53886-1MG). After coating, plates were blocked with 1% bovine serum albumin in PBS containing 0.02% Tween-20 (PBS-T) and washed with PBS-T. Undiluted culture supernatants were added to each well and incubated for 2 hours at room temperature. For detection, HRP-conjugated goat anti-IgM-Fc (1:20,000 dilution, Bethyl Laboratories, catalog no. A80-100P) or HRP-conjugated goat anti-IgG-Fc (1:10,000 dilution, Bethyl Laboratories, catalog no. A80-104P) antibodies were added separately and incubated for 2 hours at room temperature. Color was developed using 3,3′,5,5′-tetramethylbenzidine substrate (SeraCare, catalog no. 5120-0075), and the reaction was stopped with 0.2 N sulfuric acid (Fujifilm Wako Pure Chemical Corporation, catalog no. 192-04696). Absorbance was measured at 450 nm using a microplate reader (Multiskan FC, Thermo Fisher Scientific).

### RNA sequencing analysis

Total RNA was extracted from sorted IF-Tfh cells with TRIzol reagent (Thermo Fisher Scientific, catalog no. 15596018). RNA sequencing libraries were generated using the SMART-Seq v4 Ultra Low Input RNA Kit for Sequencing (Clontech, catalog no. 634888) and the Nextera XT DNA Library Preparation Kit (Illumina, catalog no. FC-131-1024). Libraries were sequenced on an Illumina NovaSeq 6000 high-throughput platform (Macrogen Japan, Tokyo, Japan). Adapter sequences and low-quality bases at read ends were removed using Trimmomatic (version 0.38). Trimmed reads were aligned to the GRCh38 reference genome with HISAT2 (version 2.1.0), and transcripts were assembled with StringTie (version 2.1.3b). Gene and transcript expression levels were quantified for each sample and reported as raw read counts and transcripts per kilobase million (TPM). Differentially expressed genes between the IgAN and RT groups were identified using DESeq2, with significance defined as *P* < 0.05. Gene set enrichment analysis (GSEA) was conducted using GSEA software (version 4.4.0; UC San Diego, La Jolla, CA), referencing Gene Ontology Biological Process (GOBP) terms and human tonsillar CD4^+^ T-cell clusters from HCA tonsil data (version 1.6.0; 25). Data visualization was performed using R software (version 4.4.3) for principal component analysis (PCA) and bubble plots, ggVolcanoR (version 1.0) for volcano plots, pheatmap (version 1.0.13) and for heatmaps.

### Statistics

Differences between 2 groups were assessed with the Mann–Whitney U test, and comparisons among 3 or more groups were performed using Dunn’s multiple-comparison test. Correlations were evaluated using Pearson or Spearman correlation coefficients, depending on variable distributions. Sample sizes and replicate numbers were determined empirically or from preliminary data to provide sufficient statistical power to detect biologically meaningful effects. All univariate statistical analyses were performed in GraphPad Prism (GraphPad Software, version 10.5.0). Random forest analysis was conducted using the randomForest package (version 4.7-1.2) with 2,000 trees. *P* < 0.05 was considered statistically significant, with significance denoted by asterisks in the figures.

## Results

### T cell subsets in IgAN tonsils

**Figure 1:**
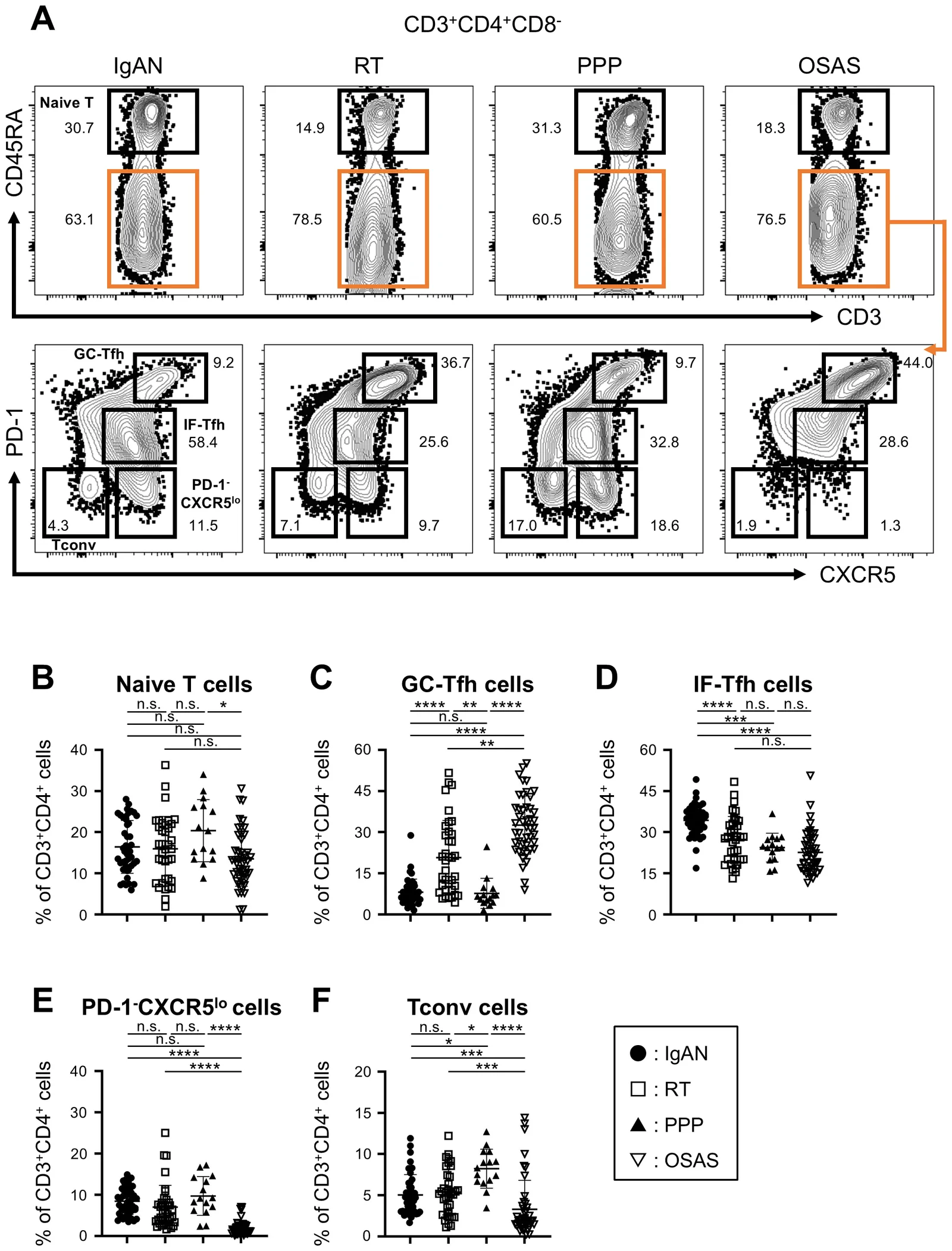
Tonsillar T-cell subsets in IgAN. **(A)** Representative flow cytometric profiles of CD4^+^ T-cell subsets in IgAN, RT, PPP, and OSAS tonsils. Numbers indicate the percentage of cells in each gate. **(B–F)** Frequencies of CD4^+^ T-cell subsets among total CD4^+^ T cells (CD3^+^CD4^+^CD8^-^) in IgAN, RT, PPP, and OSAS tonsils (see **Supplementary Table 1**). **(B)** Naive T cells (CD45RA^+^). **(C)** GC-Tfh cells (CD45RA^−^PD-1^hi^CXCR5^hi^). **(D)** IF-Tfh cells (CD45RA^−^PD-1^lo^CXCR5^lo^). **(E)** PD-1^-^CXCR5^lo^ cells (CD45RA^−^PD-1^−^CXCR5^lo^). **(F)** Tconv cells (CD45RA^−^PD-1^−^CXCR5^−^). Data are presented as mean ± SD. Statistical significance was assessed using Dunn’s multiple comparison test; *P < 0.05, **P < 0.01, ***P < 0.001, ****P < 0.0001; n.s., not significant.

We first investigated the characteristics of tonsillar T cells in patients with IgAN by flow cytometry (**Supplementary Table 1**). In parallel, we analyzed tonsillar lymphocytes from patients with recurrent tonsillitis (RT), palmoplantar pustulosis (PPP), and obstructive sleep apnea syndrome (OSAS). PPP is associated with chronic tonsillar inflammation similar to that observed in IgAN. IgAN tonsils (38.3% ± 11.8%, mean ± SE) and PPP tonsils (36.9% ± 16.1%) contained higher proportions of CD4^+^ T cells than RT (28.9% ± 13.9%) and OSAS tonsils (18.4% ± 8.2%) (**Supplementary Figures 1A**, **B**). CD8^+^ T cell frequencies were largely similar in IgAN (3.5% ± 2.2%), PPP (2.6% ± 2.4%), and RT (3.3% ± 1.8%) tonsils, but were lower in OSAS tonsils (2.0% ± 1.2%) (**Supplementary Figure 1C**). Using a gating strategy on CD4^+^ T cells (CD3^+^CD4^+^CD8^-^; **Supplementary Figure 1A**), we further delineated CD4^+^ T cell subsets, including naive CD4^+^ T cells (CD45RA^+^) and activated CD4^+^ T cells (CD45RA^-^; **Figure 1A**). Among the activated subsets, we quantified germinal center T follicular helper (GC-Tfh) cells (CD4^+^PD-1^hi^CXCR5^hi^), interfollicular Tfh (IF-Tfh) cells (CD4^+^PD-1^lo^CXCR5^lo^), CD4^+^PD-1^-^CXCR5^lo^ T cells, and conventional CD4^+^ T cells (Tconv; CD4^+^PD-1^-^CXCR5^-^; 25–27). IF-Tfh cells comprise 2 transitional populations: one progressing toward GC-Tfh cells (pre–GC-Tfh cells) and another linking GC-Tfh cells to CD4^+^PD-1^−^CXCR5^lo^ T cells, which are considered tissue counterparts of circulating Tfh cells (CD4^+^CXCR5^lo^ T cells) in peripheral blood, based on tonsillar single-cell analyses and previous studies (25, 27–29). The frequency of naive CD4^+^ T cells among total CD4^+^ T cells in IgAN tonsils (16.4% ± 6.4%) was similar to that in RT (15.9% ± 7.9%) and PPP (20.4% ± 7.6%) tonsils, but slightly higher than that in OSAS tonsils (13.5% ± 6.7%) (**Figure 1B**). With respect to germinal center activity, GC-Tfh cells were more abundant in RT (20.7% ± 13.5%) and OSAS (32.6% ± 10.6%) tonsils than in IgAN (8.1% ± 4.7%) and PPP (7.7% ± 5.5%) tonsils (**Figure 1C**). By contrast, IF-Tfh cells were markedly increased in IgAN tonsils (34.3% ± 5.7%), followed by RT (27.3% ± 8.4%), PPP (24.4% ± 5.3%), and OSAS (22.6% ± 7.7%) tonsils (**Figure 1D**). PD-1^-^CXCR5^lo^ T cells were modestly increased in IgAN (8.4% ± 3.2%) and PPP (9.7% ± 4.7%) tonsils compared with RT (7.0% ± 5.3%) and OSAS (2.0% ± 1.8%) tonsils (**Figure 1E**). These findings indicate that, in IgAN tonsils, differentiation of Tfh-related cells is, at least in part, skewed toward IF-Tfh and PD-1^−^CXCR5^lo^ phenotypes. The frequency of Tconv cells in IgAN tonsils (5.0% ± 2.5%) was comparable to that in RT tonsils (5.4% ± 2.8%) and intermediate between that in PPP (8.2% ± 2.4%) and OSAS (3.3% ± 3.5%) tonsils (**Figure 1F**).

**Figure 2:**
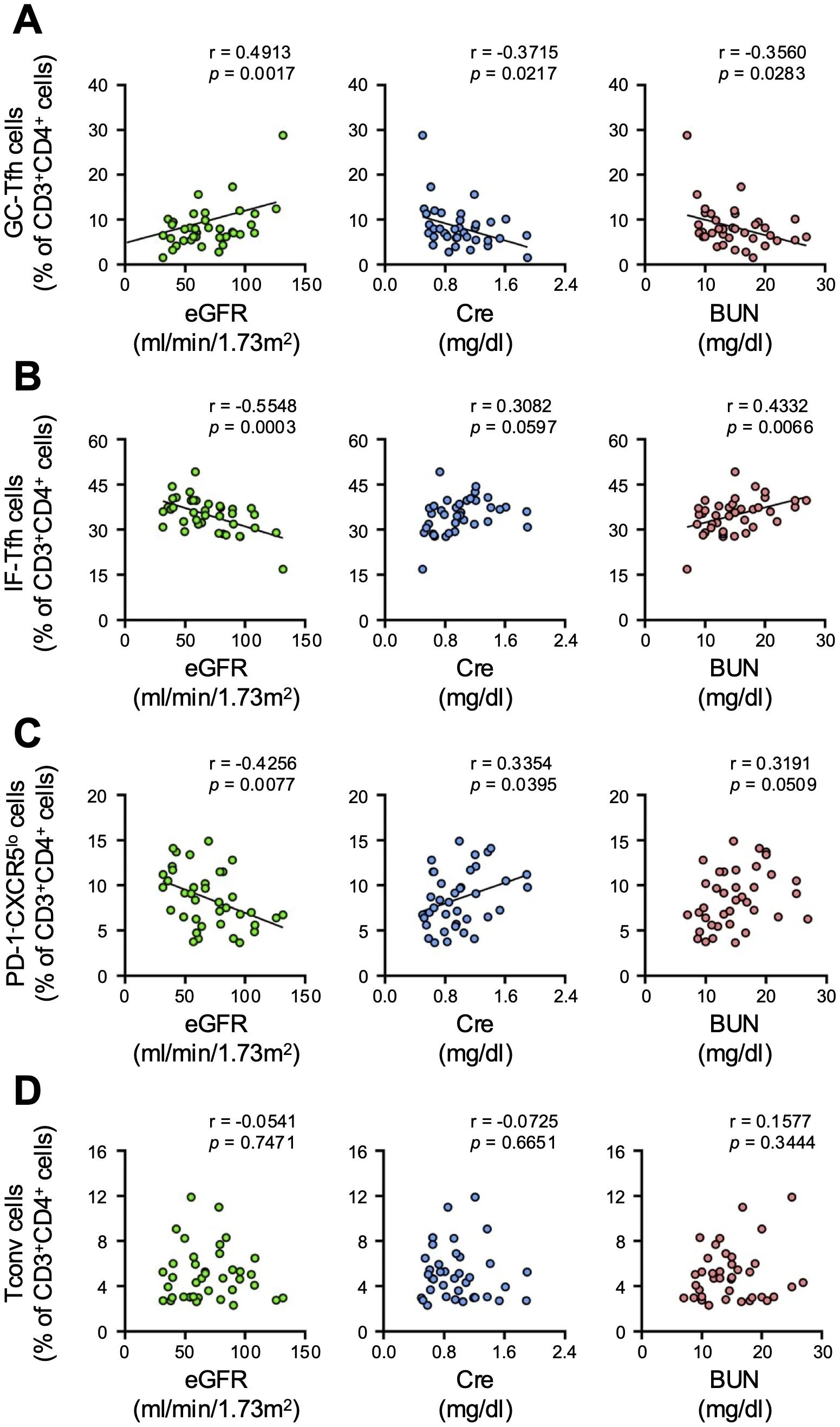
Associations between tonsillar T-cell subsets and renal function parameters in IgAN. **(A–D)** Correlation analyses between tonsillar CD4^+^ T-cell subset frequencies and renal function parameters (eGFR, creatinine, and BUN) in IgAN. **(A)** GC-Tfh cells. **(B)** IF-Tfh cells. **(C)** PD-1^-^CXCR5^lo^ cells. **(D)** Tconv cells. Statistical significance was assessed using Pearson’s correlation coefficient. Fitted linear regression lines are shown for scatter plots with *P* < 0.05.

We next examined associations between tonsillar T cell subset frequencies and renal function in patients with IgAN (**Supplementary Table 2**). GC-Tfh cell frequencies correlated positively with estimated glomerular filtration rate (eGFR; 30) and negatively with blood urea nitrogen (BUN) and creatinine, consistent with better renal function (**Figure 2A**). In contrast, higher IF-Tfh cell frequencies were associated with poorer renal function, showing negative correlations with eGFR and positive correlations with BUN and, to a lesser extent, creatinine (**Figure 2B**). Similarly, increased PD-1^−^CXCR5^lo^ cell frequencies correlated with impaired renal function, as reflected by negative correlations with eGFR and positive trends with BUN and creatinine (**Figure 2C**). Tconv cell frequencies showed no significant correlations with these renal parameters (**Figure 2D**). No such associations were observed in patients with RT (**Supplementary Figures 2A–D**). Collectively, these findings suggest that reduced GC-Tfh frequencies, together with increased IF-Tfh and PD-1^−^CXCR5^lo^ populations, shape a pathological immune environment in IgAN tonsils. Supporting this interpretation, tonsillar IF-Tfh frequencies correlated positively with proteinuria in patients with IgAN (**Supplementary Figures 3A, B**).

### B cell subsets in IgAN tonsils

**Figure 3:**
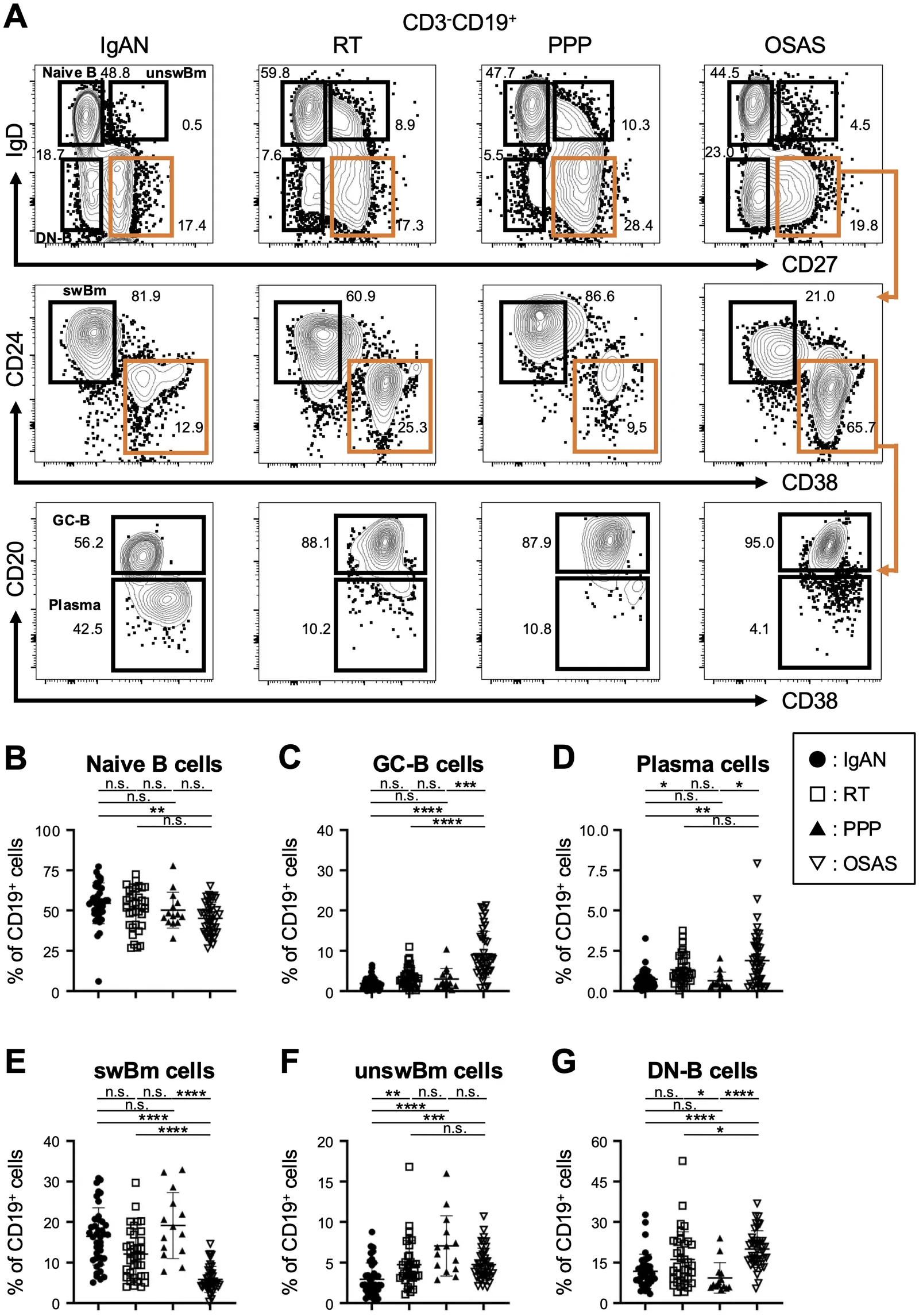
Tonsillar B-cell subsets in IgAN. **(A)** Representative flow cytometric profiles of B-cell subsets in IgAN, RT, PPP, and OSAS tonsils. Numbers indicate the percentage of cells in each gate. **(B–G)** Frequencies of B-cell subsets among total B cells (CD3^-^CD19^+^) in IgAN, RT, PPP, and OSAS tonsils. **(B)** Naive B cells (IgD^+^CD27^−^). **(C)** GC-B cells (IgD^−^CD27^+^CD24^−^CD38^+^CD20^+^). **(D)** Plasma cells (IgD^−^CD27^+^CD24^−^CD38^+^CD20^−^). **(E)** swBm cells (IgD^−^CD27^+^CD24^+^CD38^−^). **(F)** unswBm cells (IgD^+^CD27^+^). **(G)** DN-B cells (IgD^−^CD27^−^). Data are presented as mean ± SD. Statistical significance was assessed using Dunn’s multiple comparison test; *P < 0.05, **P < 0.01, ***P < 0.001, ****P < 0.0001; n.s., not significant.

In parallel with the T cell analysis, we profiled tonsillar B cell subsets by flow cytometry to investigate mechanisms of pathological antibody production (**Supplementary Table 3**). According to the gating strategy for tonsillar B cells (**Figure 3A**; **Supplementary Figure 4A**), we identified B cell subsets including naive B cells (IgD^+^CD27^−^), germinal center (GC) B cells (IgD^−^CD20^+^CD24^−^CD27^+^CD38^+^), plasma cells (IgD^−^CD20^−^CD24^−^CD27^+^CD38^+^), class-switched memory B (swBm) cells (IgD^−^CD20^+^CD24^−^CD27^+^CD38^+^), unswitched memory B (unswBm) cells (IgD^+^CD27^+^), and double-negative (DN) B cells (IgD^−^CD27^−^; 31–34). The total B cell frequency in IgAN tonsils (56.4% ± 11.5%) was comparable to that in RT (61.5% ± 10.2%) and PPP tonsils (51.9% ± 10.0%) and lower than that in OSAS tonsils (70.0% ± 10.2%) (**Supplementary Figure 4B**). Among total B cells, the proportion of naive B cells in IgAN tonsils (54.5% ± 12.7%) was similar to that in RT (51.1% ± 12.6%) and PPP (50.3% ± 11.2%) tonsils and slightly higher than that in OSAS tonsils (45.2% ± 10.0%) (**Figure 3B**). GC B cell frequencies were similar across IgAN (1.9% ± 1.5%), RT (3.4% ± 2.4%), and PPP (3.0% ± 2.6%) tonsils, but were lower than those observed in OSAS tonsils (9.2% ± 5.7%) (**Figure 3C**). Plasma cell frequencies in IgAN tonsils (0.75% ± 0.58%) were comparable to those in PPP tonsils (0.65% ± 0.52%) but were reduced relative to RT (1.33% ± 0.92%) and OSAS (1.89% ± 1.66%) tonsils (**Figure 3D**). Regarding memory B cell subsets, swBm cells were more abundant in IgAN (16.5% ± 7.1%), RT (12.1% ± 6.2%), and PPP (19.1% ± 8.1%) tonsils than in OSAS tonsils (5.9% ± 3.2%) (**Figure 3E**). In contrast, unswBm cells were decreased in IgAN tonsils (2.9% ± 2.0%) compared with RT (4.7% ± 3.0%), PPP (7.1% ± 3.7%), and OSAS (4.8% ± 2.0%) tonsils (**Figure 3F**). DN B cell frequencies were similar among IgAN (11.8% ± 6.3%), RT (16.1% ± 10.3%), and PPP (9.3% ± 5.6%) tonsils, whereas OSAS tonsils displayed a distinct profile (20.0% ± 6.8%) (**Figure 3G**). IgAN and PPP are both classified as focal tonsillar infections, particularly in the setting of immunologically mediated disease (35). Consistent with this classification, IgAN and PPP tonsils exhibited broadly similar distributions of naive, GC, plasma, swBm, and DN B cells. However, a marked reduction in unswBm cells distinguished IgAN tonsils from the other groups.

**Figure 4:**
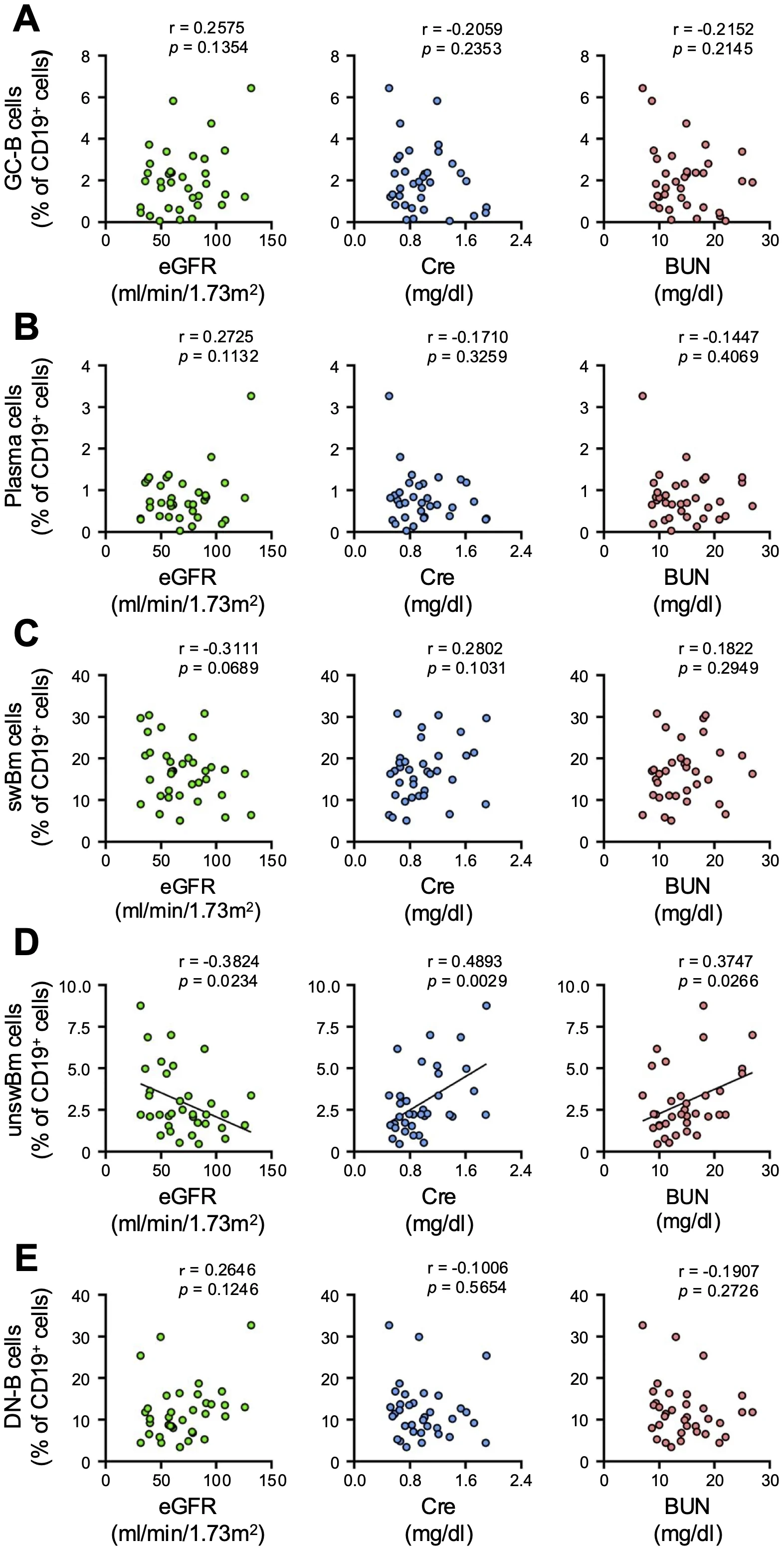
Associations between tonsillar B-cell subsets and renal dysfunction in IgAN. **(A–E)** Correlation analyses between tonsillar B-cell subset frequencies and renal function parameters (eGFR, creatinine, and BUN) in IgAN. **(A)** GC-B cells. **(B)** Plasma cells. **(C)** swBm cells. **(D)** unswBm cells. **(E)** DN-B cells. Statistical significance was assessed using Pearson’s correlation coefficient. Fitted linear regression lines are shown for scatter plots with *P* < 0.05.

We further examined associations between tonsillar B cell subsets and renal function in patients with IgAN (**Supplementary Table 4**). UnswBm cell frequencies showed distinct relationships with serum markers, with a negative correlation with eGFR and positive correlations with BUN and creatinine (**Figure 4D**). In contrast, no significant correlations were detected between other B cell subsets and these renal parameters (**Figures 4A–C, E**). In RT patients, analogous analyses revealed no correlations between tonsillar B cell subsets and clinical parameters (**Supplementary Figures 5A–E**). B cell subset frequencies were also unrelated to urinary findings, including proteinuria and hematuria (**Supplementary Figures 6A, B**). Together, these results suggest that reduced unswBm cell frequencies may contribute to renal dysfunction in IgAN, even in the absence of overt urinary abnormalities.

### Pathological immune settings in IgAN tonsils

**Figure 5:**
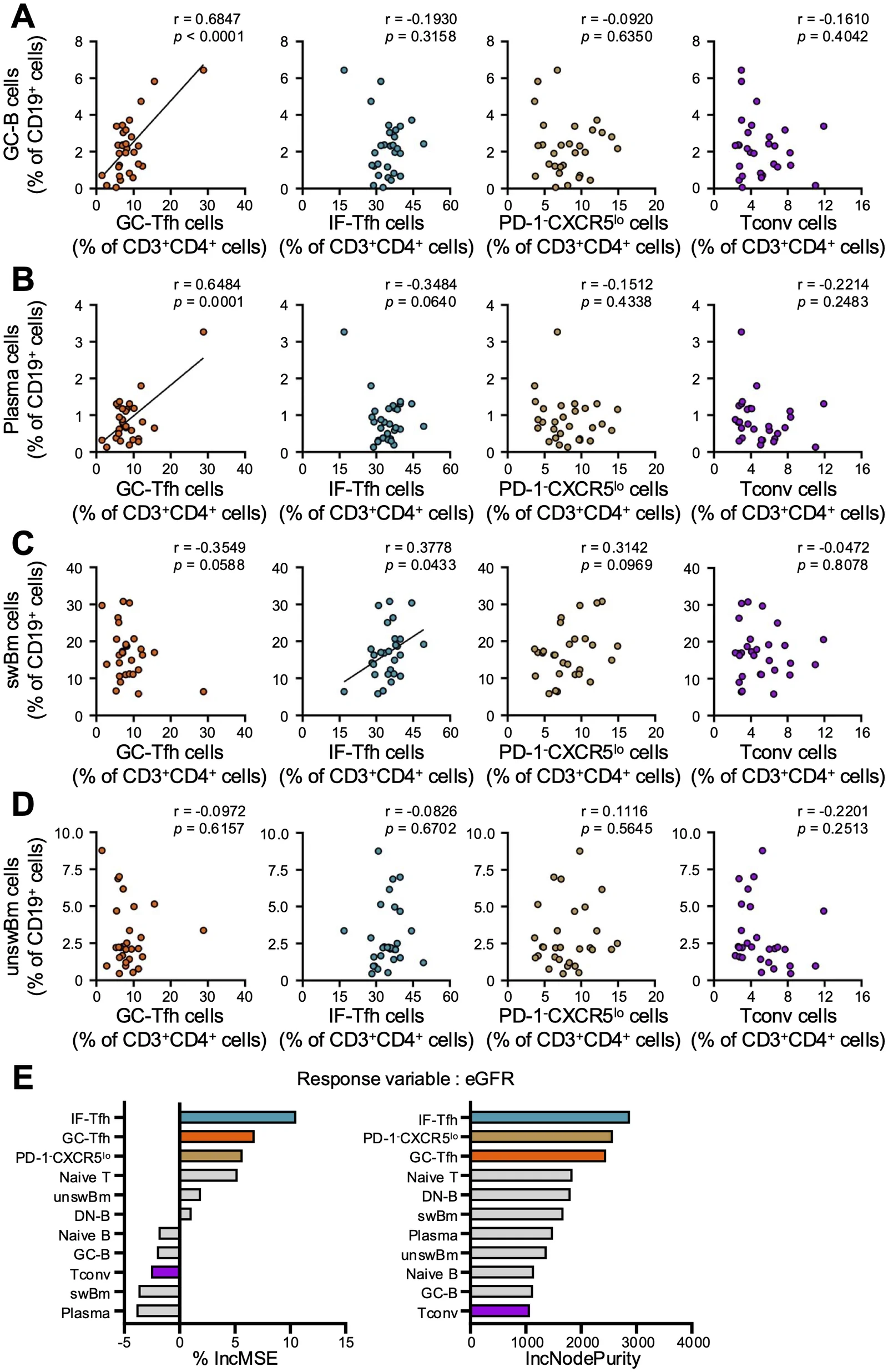
Correlations between tonsillar T- and B-cell subset frequencies in IgAN. **(A–D)** Correlation analyses of T- and B-cell subset frequencies in IgAN tonsils. **(A)** GC-B cells. **(B)** Plasma cells. **(C)** swBm cells. **(D)** unswBm cells. **(E)** Random forest analysis with eGFR as the response variable. The plots show the percent increase in mean squared error (%IncMSE) and the increase in node purity (IncNodePurity) in the left and right panels, respectively. Statistical significance was assessed using Pearson’s correlation coefficient. Fitted linear regression lines are shown for scatter plots with *P* < 0.05.

Building on the analysis of tonsillar T and B cell subsets in relation to renal abnormalities, we next examined their interrelationships to clarify immune mechanisms underlying IgAN, using RT tonsils as a comparator (**Supplementary Table 5**). In IgAN tonsils, GC-Tfh cell frequencies, but not those of other T cell subsets, were significantly correlated with GC B cell and plasma cell frequencies (**Figures 5A, B**). Similar patterns were observed in RT tonsils (**Supplementary Figures 7A, B**), indicating that GC-Tfh–B cell interactions are a shared feature of IgAN and RT tonsils. In contrast, IF-Tfh cell frequencies correlated specifically with swBm cells in IgAN tonsils (**Figure 5C**), whereas in RT tonsils, GC-Tfh and PD-1^−^CXCR5^lo^ cells were more strongly associated with swBm cells (**Supplementary Figure 7C**). No correlations were detected between any T cell subset and unswBm cells in either IgAN or RT tonsils (**Figure 5D**; **Supplementary Figure 7D**). Collectively, these findings suggest that IF-Tfh–mediated immune activity may play a central role in the pathogenic mechanisms of IgAN tonsils.

After these analyses, we applied random forest modeling to identify variables associated with renal dysfunction. This algorithm ranks predictors by feature importance, providing a robust framework for characterizing tonsillar cell populations relevant to IgAN pathogenesis (36). Using eGFR as the response variable within the lymphocyte dataset from IgAN tonsils, IF-Tfh cells consistently emerged as the top-ranked feature according to both IncMSE and IncNodePurity metrics (**Figure 5E**). These findings highlight the central role of IF-Tfh cells in shaping the pathological immune landscape of IgAN. GC-Tfh and PD-1^−^CXCR5^lo^ cells also showed high importance scores, supporting their potential involvement in disease progression. Random forest analysis further indicated a modest contribution of DN B cells, although the functional relevance of this subset in IgAN remains unclear (37). Similar association patterns were observed in both IgAN and RT tonsils, where IF-Tfh cells were inversely correlated with DN B cells (**Supplementary Figures 8A, B**). Together, these results suggest that IF-Tfh–driven immune activity contributes to the distinct immunological milieu characteristic of IgAN tonsils.

### Memory B cell activation by IF-Tfh cells in IgAN tonsils

**Figure 6:**
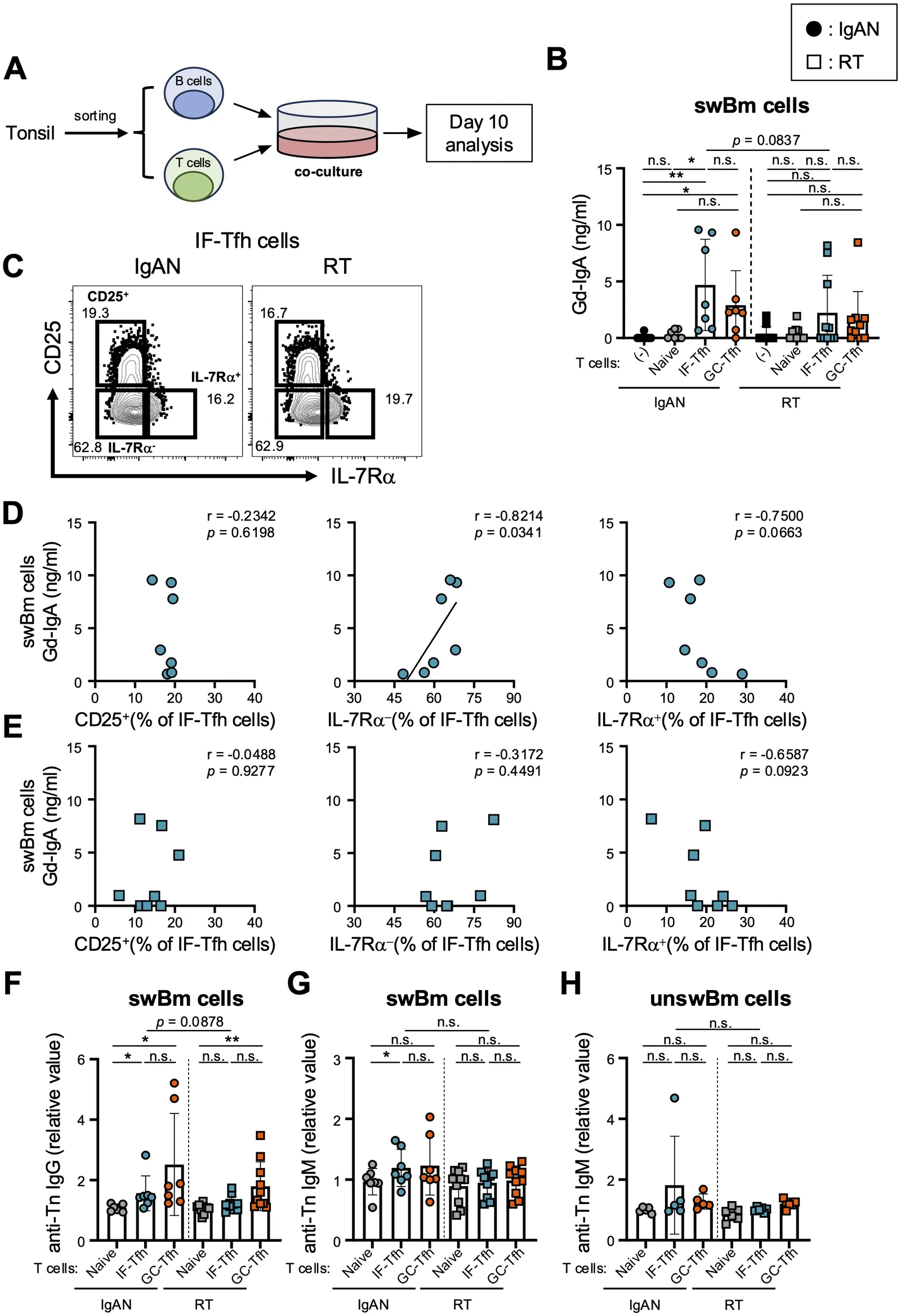
Pathological antibody production by memory B cells under IF-Tfh cell help in IgAN tonsils. **(A)** Schematic of the experimental protocol. **(B)** Levels of Gd-IgA produced by swBm cells cocultured with naive CD4^+^ T cells, IF-Tfh cells, or GC-Tfh cells (IgAN tonsils, n = 7; RT tonsils, n = 10). **(C)** Representative flow cytometric profiles of IF-Tfh cell subsets from IgAN and RT tonsils showing Treg-like (CD25^+^IL-7Rα^-^), Tem-like (CD25^-^IL-7Rα^-^), and Tcm-like (CD25^-^IL-7Rα^+^) subsets. **(D, E)** Correlation analyses showing relationships between IF-Tfh cell subset frequencies and Gd-IgA production by swBm cells. **(D)** IgAN tonsils (n = 7). **(E)** RT tonsils (n = 8). **(F, G)** Levels of anti-Tn antibodies produced by swBm cells cocultured with naive CD4^+^ T cells, IF-Tfh cells, or GC-Tfh cells (IgAN tonsils, n = 7; RT tonsils, n = 10). **(F)** Anti-Tn IgG. **(G)** Anti-Tn IgM. **(H)** Anti-Tn IgM production by unswBm cells cocultured with naive CD4^+^ T cells, IF-Tfh cells, or GC-Tfh cells (IgAN, n = 5; RT, n = 6). **(B)**, **(F–H)** Statistical significance within each disease group was assessed using Dunn’s multiple comparison test, and comparisons of IF-Tfh cells between IgAN and RT were performed using the Mann–Whitney test. *P < 0.05, **P < 0.01; n.s., not significant. **(D, E)** Statistical significance was assessed using Spearman’s correlation coefficient. Fitted linear regression lines are shown for scatter plots with *P* < 0.05.

Next, we conducted *in vitro* coculture experiments using autologous tonsillar cells to examine the functional role of IF-Tfh cells in regulating antibody production (**Supplementary Table 6**). We focused on interactions between IF-Tfh cells and memory B cell subsets, specifically swBm and unswBm cells, based on our preceding observations. Using established protocols (**Figure 6A**; **Supplementary Figures 9A, B**), we found that IF-Tfh cells from IgAN tonsils promoted Gd-IgA production by swBm cells, whereas this effect was not observed in RT tonsils (**Figure 6B**). Because IF-Tfh cells comprise heterogeneous T cell subsets, we further studied tonsillar IF-Tfh cells by assessing the conventional markers CD25 and CD127 (IL-7Rα), identifying three phenotypic subsets: T follicular regulatory (Tfr)–like (CD25^+^CD127^−^), T effector memory (Tem)–like (CD25^−^CD127^−^), and T central memory (Tcm)–like (CD25^−^CD127^+^) IF-Tfh cells (**Figure 6C**; 38, 39). Notably, the frequency of Tem-like IF-Tfh cells in IgAN tonsils showed a strong positive correlation with Gd-IgA production by swBm cells, whereas Tfr-like and Tcm-like subsets did not (**Figure 6D**). In contrast, none of these IF-Tfh subsets in RT tonsils correlated with Gd-IgA production (**Figure 6E**). For anti-Tn antibody production, IF-Tfh cells enhanced anti-Tn IgG and IgM secretion by swBm cells in IgAN tonsils, an effect that was absent in RT tonsils (**Figures 6F, G**). By contrast, anti-Tn IgM production by unswBm cells was not affected by any T cell subset under either condition (**Figure 6H**). Taken together, these findings suggest that IF-Tfh cells may contribute to IgAN pathogenesis by promoting Gd-IgA–related immune complex formation through their interaction with swBm cells in the tonsils.

### Gene expression signatures of IF-Tfh cells in IgAN tonsils

**Figure 7:**
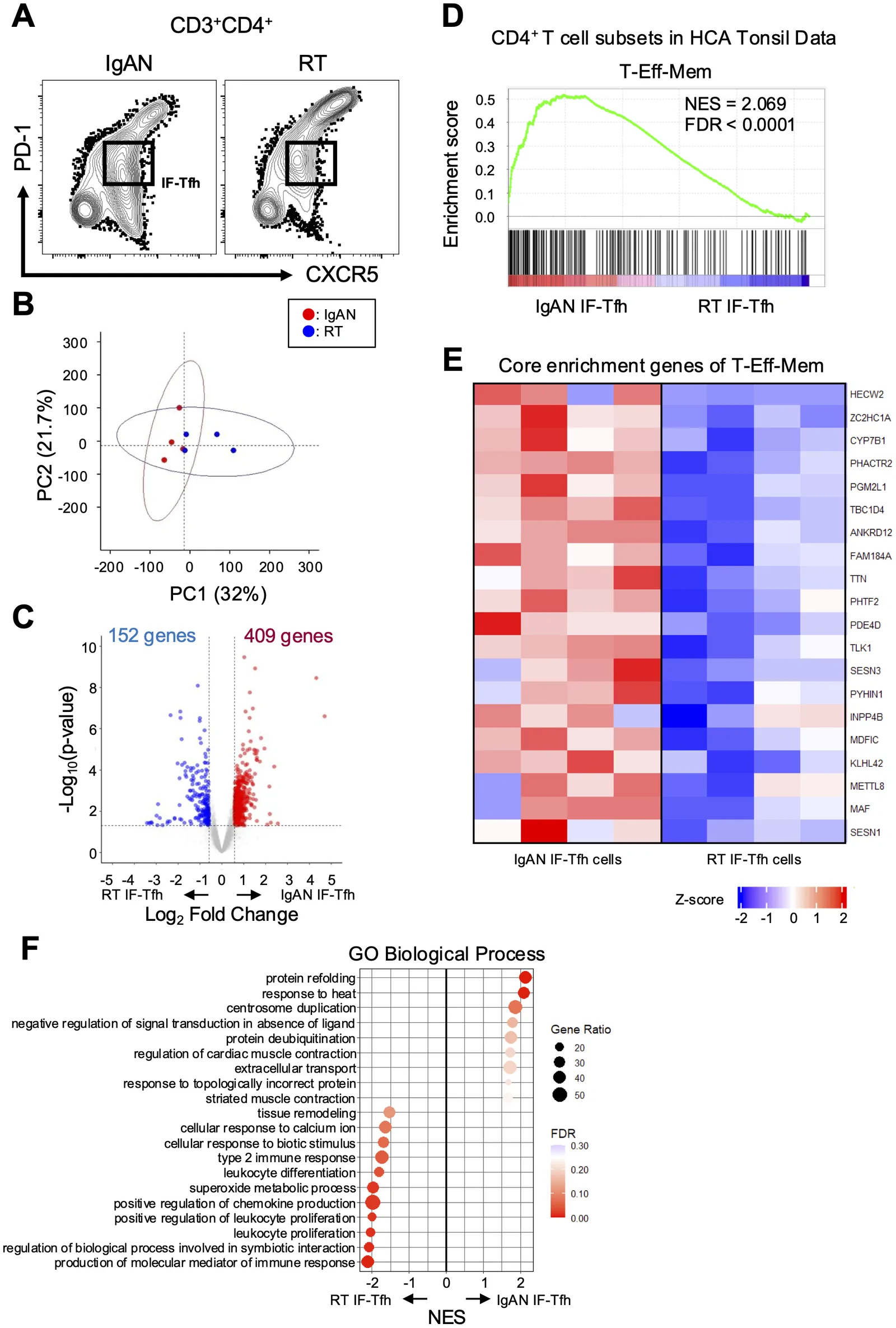
Gene expression profiling of tonsillar IF-Tfh cells in IgAN. **(A)** Flow cytometric sorting strategy for IF-Tfh cells. **(B)** Principal component analysis (PCA) of bulk RNA-seq data from IF-Tfh cells isolated from IgAN tonsils (red) and RT tonsils (blue). Ellipses indicate 95% confidence intervals. **(C)** Volcano plot showing differentially expressed genes (*P* < 0.05) with more than 1.5-fold change (log_2_ fold change > 0.58 or < −0.58) in IgAN IF-Tfh cells compared with RT IF-Tfh cells. **(D)** Gene set enrichment analysis of transcriptomes from IgAN versus RT IF-Tfh cells based on CD4^+^ T-cell clusters in the HCA tonsil dataset. Gene sets with a false discovery rate (FDR) < 0.25 are shown. NES, normalized enrichment score. **(E)** Heatmap showing expression profiles of core enrichment genes identified in **(D)** for IgAN and RT IF-Tfh cells. **(F)** Gene ontology analysis of biological processes and NES values in IgAN versus RT IF-Tfh cells.

We further characterized the transcriptomic features of tonsillar IF-Tfh cells in patients with IgAN and RT (**Supplementary Table 7**) by performing comparative RNA-seq analysis of isolated IF-Tfh cells (**Figure 7A**; **Supplementary Figure 10A**). Principal component analysis (PCA; PC1 versus PC2) showed that IF-Tfh cells from IgAN tonsils, although partially overlapping with those from RT tonsils, remained transcriptionally distinct (**Figure 7B**; **Supplementary Figures 10B, C**). Consistent with this divergence, volcano plot analysis identified 409 upregulated and 152 downregulated genes in IgAN-derived IF-Tfh cells (**Figure 7C**; **Supplementary Table 8**). Gene set enrichment analysis (GSEA) of helper CD4^+^ T cell subsets in tonsils, based on normalized enrichment scores (NES), showed that IgAN IF-Tfh cells shared transcriptomic features with Tem cells (**Figures 7D, E**; **Supplementary Table 9**; 25). IgAN IF-Tfh cells were also enriched for Gene Ontology (GO) terms related to protein refolding, heat response, centrosome duplication, negative regulation of ligand-independent signaling, and protein deubiquitination (**Figure 7F**). These pathways indicate that IgAN IF-Tfh cells are stress-adapted, proliferative, and metabolically active, with altered signaling and regulatory control. By contrast, IF-Tfh cells from RT tonsils displayed transcriptional profiles that partially resembled naive CD4^+^ T cells. Together, these findings suggest that IF-Tfh cells in IgAN tonsils may constitute a heterogeneous population enriched for Tem-like features, distinct from those observed in RT tonsils.

### Circulating CD4^+^ T cells reflecting tonsillar immune activity in IgAN

**Figure 8:**
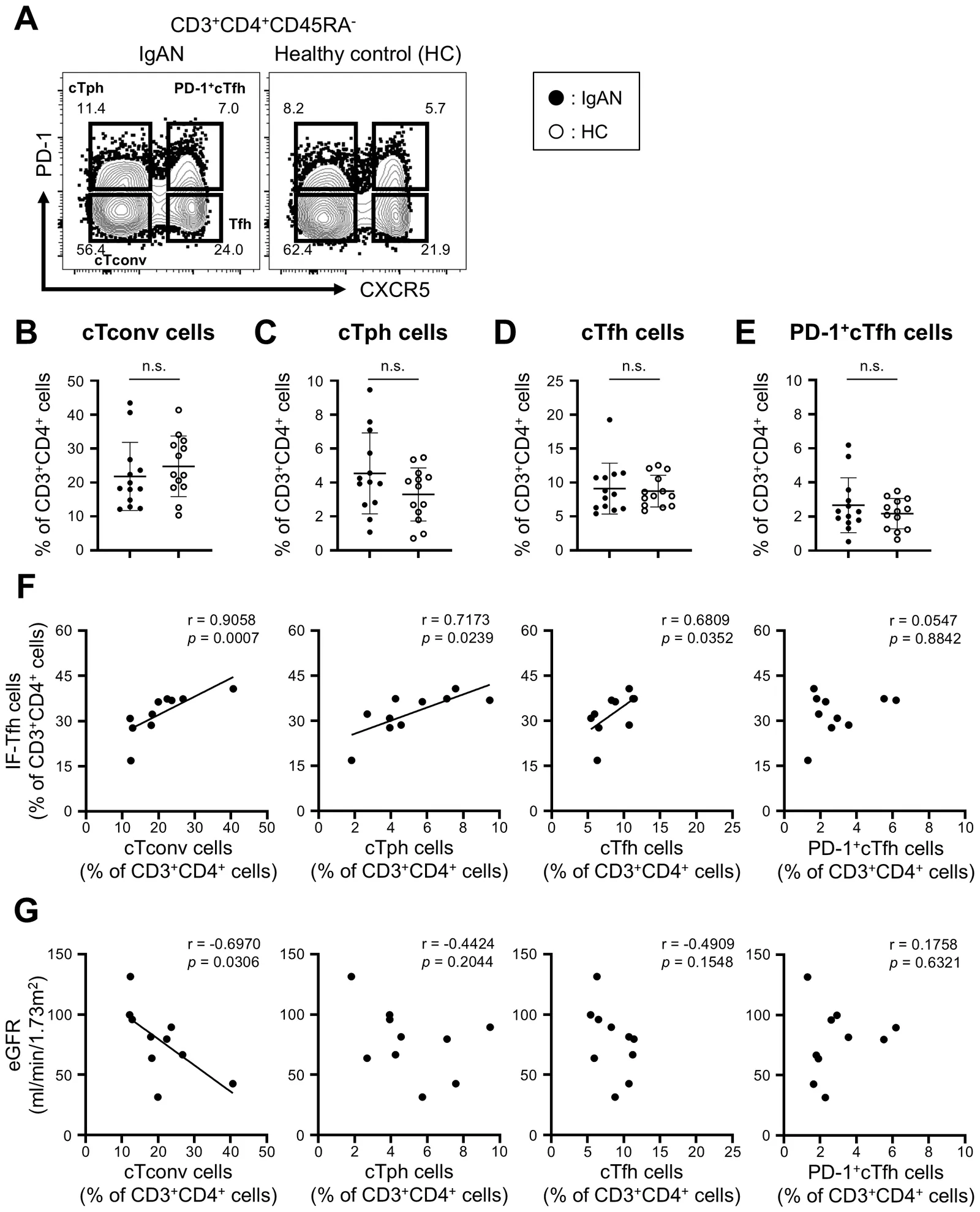
Correlations between tonsillar IF-Tfh cells and circulating CD4^+^ T-cell subsets in IgAN. **(A)** Representative flow cytometric profiles of CD3^+^CD4^+^CD45RA^-^ T-cell subsets in peripheral blood from patients with IgAN and healthy donor controls (HC). **(B–E)** Frequencies of circulating CD4^+^ T-cell subsets in IgAN and HC (IgAN, n = 12; HC, n = 13). **(B)** cTconv cells (PD-1^−^CXCR5^−^). **(C)** cTph cells (PD-1^+^CXCR5^-^). **(D)** cTfh cells (PD-1^-^CXCR5^+^). **(E)** PD-1^+^ cTfh cells (PD-1^+^CXCR5^+^). **(F)** Scatter plots and correlation coefficients showing the relationships between IF-Tfh cell frequency within tonsillar CD4^+^ T cells and the frequencies of cTconv, cTph, cTfh, and PD-1^+^ cTfh cells within peripheral blood CD4^+^ T cells in IgAN (n = 9). **(G)** Scatter plots and correlation coefficients showing the relationships between eGFR and the frequencies of cTconv, cTph, cTfh, and PD-1^+^ cTfh cells within peripheral blood CD4^+^ T cells in IgAN (n = 9). **(B–E)** Statistical significance was assessed using Mann–Whitney U tests. n.s., not significant. **(F, G)** Statistical significance was assessed using Spearman correlation. Approximate linear regression lines are shown when *P* < 0.05.

Finally, we examined how tonsillar IF-Tfh cells relate to circulating CD4^+^ T-cell subsets in patients with IgAN (**Supplementary Table 10**). Based on PD-1 and CXCR5 expression, activated peripheral CD4^+^ T cells (CD3^+^CD4^+^CD45RA^−^) were subdivided into circulating conventional T cells (cTconv, PD-1^−^CXCR5^−^), circulating peripheral helper T cells (cTph, PD-1^+^CXCR5^−^), circulating follicular helper T cells (cTfh, PD-1^−^CXCR5^+^), and circulating PD-1^+^ follicular helper T cells (cPD-1^+^Tfh, PD-1^+^CXCR5^+^) (**Figure 8A**; **Supplementary Figure 11A**). The frequencies of these CD4^+^ T-cell subsets in patients with IgAN were similar to those in healthy controls, consistent with comparable levels of total and naive CD4^+^ T cells (**Figures 8B–E**, **Supplementary Figures 11B, C**). Notably, tonsillar IF-Tfh cell frequencies were positively correlated with frequencies of cTconv, cTph, and cTfh cells, but not PD-1^+^ cTfh cells, in patients with IgAN (**Figure 8F**). When circulating CD4^+^ T-cell subsets were related to renal function parameters, cTconv cell frequencies showed a strong inverse correlation with eGFR, whereas cTph, cTfh, and PD-1^+^ cTfh cells showed no significant associations (**Figure 8G**). Taken together, these findings indicate that cTconv cell frequency may reflect tonsillar IF-Tfh cell activity associated with renal dysfunction. However, because IF-Tfh and Tconv cell frequencies in IgAN tonsils were not correlated (**Supplementary Figure 11D**), tonsillar Tconv cells are likely distinct from those circulating in the peripheral blood.

## Discussion

Studies of the mechanisms underlying IgAN pathogenesis have largely focused on dysregulated IgA, a principal mediator of mucosal immunity. In this context, we examined tonsils from patients with IgAN as key sites of IgA production, with particular attention to the involvement of Tfh cells in regulating humoral immune responses (22, 26). Our data indicate that a marked elevation of IF-Tfh cells is a distinguishing feature of IgAN tonsils compared with tonsils from RT, PPP, and OSAS. IF-Tfh cells with an effector memory–like phenotype were also substantially increased in IgAN tonsils and may contribute to a pathological local immune milieu. Among B-cell subsets, swBm cells showed a specific association with IF-Tfh cells in promoting Gd-IgA and anti-Tn antibody production within IgAN tonsils, consistent with coordinated cellular interactions that drive dysregulated antibody responses and facilitate the formation of nephritogenic immune complexes. Because elevated serum Gd-IgA1 levels correlate with poor prognosis in IgAN, IF-Tfh cells in IgAN tonsils may represent a potential target for modulating Gd-IgA production (40). Although the precise localization of IF-Tfh cell–swBm cell interactions within the tonsils remains unclear, such interactions are likely to occur within interfollicular or perifollicular regions, where memory B cells commonly encounter T cells (41, 42). Further elucidation of this process may provide important insights for developing strategies to more effectively target IF-Tfh cells in IgAN. IF-Tfh–driven memory B cell activation identified in tonsillar tissue may represent a shared mucosal immune mechanism that operates across both NALT and GALT, potentially linking local mucosal immune dysregulation to systemic production of nephritogenic Gd-IgA.

Accumulating evidence indicates that IF-Tfh cells (CD4^+^PD-1^lo^CXCR5^lo^) constitute a heterogeneous population that includes both pre-Tfh cells, which retain the potential to differentiate into mature GC-Tfh cells, and transitional Tfh cells that arise from GC-Tfh cells as they exit germinal centers during the contraction phase of the immune response (25, 27–29). Beyond this developmental continuum, GC-Tfh cells are thought to persist even after germinal center collapse and to maintain distinct molecular features that set them apart from conventional memory CD4^+^ T cells (43). As these GC-Tfh cells transition into memory Tfh cells, they progressively downregulate PD-1. The mechanisms responsible for the marked elevation of IF-Tfh cells in IgAN tonsils have yet to be elucidated; however, current evidence points toward a contribution from the memory IF-Tfh cell compartment. Effector memory T cells are generally characterized by their long-lived nature, intrinsic capacity for self-renewal, and ability to persist in the absence of continuous antigenic stimulation (44). These attributes raise the possibility that a portion of the IF-Tfh cell pool in IgAN tonsils may be sustained through memory-associated processes. Furthermore, PD-1^lo^ Tfh cells within the Tfh-cell lineage are thought to represent a proliferative and metabolically active phase. Thus, IF-Tfh cells in IgAN tonsils may encompass an expanded reservoir of proliferating, memory-biased Tfh cells that accumulate over time in response to chronic or dysregulated mucosal immune activation. This model supports the concept that an altered immunological environment in IgAN tonsils could drive repeated cycles of Tfh activation and memory formation, thereby contributing to the enlarged IF-Tfh cell compartment and its potential role in pathological IgA responses.

Given the critical role of IF-Tfh cell activity in IgAN tonsils, we further examined circulating CD4^+^ T-cell subsets that might mirror tonsillar IF-Tfh cell activity in IgAN. Unexpectedly, tonsillar IF-Tfh cells exhibited broad positive correlations with cTconv, cTph, and cTfh cells in the peripheral blood of patients with IgAN, indicating coordinated alterations across circulating T-cell compartments. Nevertheless, among these subsets, only cTconv cells demonstrated a significant inverse correlation with eGFR, suggesting that cTconv cells may uniquely reflect renal impairment despite their shared association with tonsillar IF-Tfh cells. The cTconv cell population represents the functional backbone of peripheral CD4^+^ T-cell immunity, encompassing quiescent naive cells, central and effector memory pools, and major nonfollicular effector helper subsets, including Th1, Th2, and Th17 cells, as well as immunoregulatory Treg cells (45, 46). Given this heterogeneity, cTconv cells in patients with IgAN may contain a pathological CD4^+^ T-cell component that contributes to renal dysfunction. Thus, cTconv cells may serve as a novel indicator of IgAN pathology alongside previously identified molecular biomarkers for the disease (47). Moreover, further investigation of circulating Tfh-lineage cells in IgAN may provide additional insights into its immunopathogenesis.

Comprehensive analyses of B-cell subsets revealed that unswBm cells were associated with renal dysfunction in IgAN, even in the absence of overt urinary abnormalities. UnswBm cells are antigen-experienced B cells that express the classical memory marker CD27 while retaining IgM/IgD expression, indicating that they have not undergone class-switch recombination (48). No correlations were found between the frequencies of unswBm cells and any T-cell subset in either IgAN or RT tonsils, suggesting that their contribution to disease progression may operate independently of T-cell–mediated regulation. Consistent with this observation, we were unable to detect a positive interaction between IF-Tfh cells and unswBm cells, despite the potential association suggested by coculture experiments. UnswBm cells are known to play a critical role in IgM-mediated memory responses, raising the possibility that the anti-Tn IgM detected in patients with IgAN may be produced, at least in part, by tonsillar unswBm cells (7). Although we did not detect anti-Tn IgM derived from unswBm cells in IgAN tonsils under our experimental conditions, further investigation will be required to test this hypothesis.

In summary, our findings highlight IF-Tfh cells as key drivers linking tonsillar immune dysregulation to clinical abnormalities in IgAN. Their expansion, interactions with swBm cells, and associations with circulating CD4^+^ T-cell subsets indicate that tonsillar immune activity contributes to pathogenic IgA responses and renal dysfunction. The distinct relationship between cTconv cells and eGFR, together with the independent involvement of unswBm cells, further underscores the complex cellular pathways driving IgAN pathogenesis. These insights refine our understanding of IgAN and identify potential immunological targets for future diagnostic and therapeutic strategies.

## Data Availability

RNA sequencing data generated in this study have been deposited in the Gene Expression Omnibus (GEO) repository hosted by the National Center for Biotechnology Information (NCBI) under accession number GSE315711. The original contributions presented in the study are included in the article/Supplementary Material. Further inquiries can be directed to the corresponding author.

## Supplementary material

Supplementary Figure 1 Gating strategy for tonsillar T cells corresponding to **Figure 1**. **(A)** Representative flow cytometric profiles of tonsillar T cells (CD3^+^). **(B, C)** Graphs showing frequencies of T cells in IgAN, RT, PPP, and OSAS tonsils (**Supplementary Table 1**). **(B)** CD4^+^ T cells (CD3^+^CD4^+^). (C) CD8^+^ T cells (CD3^+^CD8^+^). Data are shown as mean ± SD. Statistical significance was assessed using Dunn’s multiple-comparison test; **P* < 0.05, ***P* < 0.01, *****P* < 0.0001; n.s., not significant.

Supplementary Figure 2 Tonsillar T-cell subsets associated with renal function parameters in RT corresponding to **Figure 2**. **(A–D)** Correlation analyses between frequencies of tonsillar CD4^+^ T-cell subsets and renal function parameters (eGFR, creatinine, and BUN) in RT (**Supplementary Table 2**). **(A)** GC-Tfh cells. **(B)** IF-Tfh cells. **(C)** PD-1^-^CXCR5^lo^ T cells. **(D)** Tconv cells. Statistical significance was assessed using Pearson’s correlation coefficient. Approximate linear regression lines are shown when *P* < 0.05.

Supplementary Figure 3 Tonsillar T-cell subsets associated with urinalysis findings in IgAN corresponding to **Figure 2**. **(A, B)** Tonsillar CD4^+^ T-cell subsets stratified by qualitative urinalysis findings in patients with IgAN (**Supplementary Table 2**). **(A)** Qualitative urine protein levels. **(B)** Qualitative urinary red blood cell levels. Data are shown as mean ± SD. Statistical significance was assessed using Dunn’s multiple-comparison test; **P* < 0.05, ***P* < 0.01, *****P* < 0.0001; n.s., not significant.

Supplementary Figure 4 Gating strategy for tonsillar B cells corresponding to **Figure 3**. **(A)** Representative flow cytometric profiles of tonsillar B cells (CD3^-^CD19^+^). **(B)** Graphs showing frequencies of B cells in IgAN, RT, PPP, and OSAS tonsils (**Supplementary Table 3**). Data are shown as mean ± SD. Statistical significance was assessed using Dunn’s multiple-comparison test; **P* < 0.05, ***P* < 0.01, *****P* < 0.0001; n.s., not significant.

Supplementary Figure 5 Tonsillar B-cell subsets associated with renal function parameters in RT corresponding to **Figure 4**. **(A–E)** Correlation analyses between frequencies of tonsillar B-cell subsets and renal function parameters (eGFR, creatinine, and BUN) in RT (**Supplementary Table 4**). **(A)** GC-B cells. **(B)** Plasma cells. **(C)** swBm cells. **(D)** unswBm cells. **(E)** DN-B cells. Statistical significance was assessed using Pearson’s correlation coefficient. Approximate linear regression lines are shown when *P* < 0.05.

Supplementary Figure 6 Tonsillar B-cell subsets associated with urinalysis findings in IgAN corresponding to **Figure 4**. **(A, B)** Tonsillar B-cell subsets stratified by qualitative urinalysis findings in patients with IgAN (**Supplementary Table 4**). **(A)** Qualitative urine protein levels. **(B)** Qualitative urinary red blood cell levels. Data are shown as mean ± SD. Statistical significance was assessed using Dunn’s multiple-comparison test.

Supplementary Figure 7 Correlations between tonsillar T-cell and B-cell subsets in RT corresponding to **Figure 5**. **(A–D)** Correlation analyses of T-cell and B-cell subset frequencies in RT tonsils (**Supplementary Table 5**). **(A)** GC-B cells. **(B)** Plasma cells. **(C)** swBm cells. **(D)** unswBm cells. Statistical significance was assessed using Pearson’s correlation coefficient. Approximate linear regression lines are shown when *P* < 0.05.

Supplementary Figure 8 Correlations between T-cell subsets and DN-B cells in tonsils corresponding to **Figure 5**. **(A, B)** Correlation analyses of T-cell subset frequencies and DN-B cell frequencies in tonsils (**Supplementary Table 5**). **(A)** IgAN tonsils. **(B)** RT tonsils. Statistical significance was assessed using Pearson’s correlation coefficient. Approximate linear regression lines are shown when *P* < 0.05.

Supplementary Figure 9 Gating strategy for sorting autologous B-cell and T-cell subsets in tonsils for coculture experiments corresponding to **Figure 6**. **(A)** Representative flow cytometric profiles of memory B-cell subsets, including swBm and unswBm cells, after magnetic bead–based selection of CD19^+^ cells from tonsillar lymphocytes. **(B)** Representative flow cytometric profiles of tonsillar CD4^+^ T-cell subsets, including naive T cells, IF-Tfh cells, and GC-Tfh cells, after magnetic bead–based depletion of CD19^+^ cells.

Supplementary Figure 10 Gating strategy for sorting tonsillar IF-Tfh cells and PCA scores corresponding to **Figure 7**. **(A)** Representative flow cytometric profiles of tonsillar CD4^+^ T cells. **(B, C)** PCA score plots from RNA-seq data of IF-Tfh cells from IgAN and RT tonsils (**Supplementary Table 6**). **(B)** PC1 scores. **(C)** PC2 scores. Statistical significance was assessed using the Mann-Whitney U test; **P* < 0.05; n.s., not significant.

Supplementary Figure 11 Gating strategy for circulating CD4^+^ T-cell subsets (corresponding to **Figure 8**) and correlations between IF-Tfh cells and other tonsillar T-cell subsets in IgAN (corresponding to **Figure 1**). **(A)** Representative flow cytometric profiles of peripheral blood mononuclear cells (PBMCs) corresponding to **Figure 8**. **(B, C)** Frequencies of CD4^+^ T cells in the peripheral blood of patients with IgAN and healthy controls (HC) (**Supplementary Table 10**). (B) Total CD4^+^ T cells (CD4^+^). **(C)** Naive CD4^+^ T cells (CD4^+^CD45RA^+^). **(D)** Correlations between IF-Tfh cell frequencies and other tonsillar T-cell subset frequencies, corresponding to **Figure 1**. **(B, C)** Statistical significance was assessed using the Mann-Whitney U test; n.s., not significant. **(D)** Statistical significance was assessed using Pearson’s correlation coefficient. Approximate linear regression lines are shown where *P* < 0.05.
